# Structure and enzymatic accessibility of leaf and stem from wheat straw before and after hydrothermal pretreatment

**DOI:** 10.1186/1754-6834-7-74

**Published:** 2014-05-20

**Authors:** Heng Zhang, Lisbeth G Thygesen, Kell Mortensen, Zsófia Kádár, Jane Lindedam, Henning Jørgensen, Claus Felby

**Affiliations:** 1 Department of Geosciences and Natural Resource Management, Faculty of Sciences, University of Copenhagen, Rolighedsvej 23, DK-1958 Frederiksberg C, Denmark; 2 Niels Bohr Institute, University of Copenhagen, Universitetsparken 5, D-03-3-06, DK-2100 Copenhagen Ø, Denmark; 3 Center for BioProcess Engineering, Department of Chemical and Biochemical Engineering, Technical University of Denmark, DTU, DK-2800, Kgs, Lyngby, Denmark; 4 Department of Plant and Environmental Sciences, University of Copenhagen, Thorvaldsensvej 40, DK-1871 Frederiksberg C, Denmark; 5 Department of Chemical and Biochemical Engineering, Technical University of Denmark, 2800 Kgs, Lyngby, Denmark

**Keywords:** Wheat straw anatomical fractions, recalcitrance, water, cellulose crystallinity, glucan accessibility

## Abstract

**Background:**

Biomass recalcitrance is affected by a number of chemical, physical and biological factors. In this study we looked into the differences in recalcitrance between two major anatomical fractions of wheat straw biomass, leaf and stem. A set of twenty-one wheat cultivars was fractionated and illustrated the substantial variation in leaf-to-stem ratio between cultivars. The two fractions were compared in terms of chemical composition, enzymatic convertibility, cellulose crystallinity and glucan accessibility. The use of water as a probe for assessing glucan accessibility was explored using low field nuclear magnetic resonance and infrared spectroscopy in combination with hydrogen-deuterium exchange.

**Results:**

Leaves were clearly more degradable by lignocellulolytic enzymes than stems, and it was demonstrated that xylose removal was more linked to glucose yield for stems than for leaves. Comparing the locations of water in leaf and stem by low field NMR and FT-IR revealed that the glucan hydroxyl groups in leaves were more accessible to water than glucan hydroxyl groups in stems. No difference in crystallinity between leaf and stem was observed using wide angle x-ray diffraction. Hydrothermal pretreatment increased the accessibility towards water in stems but not in leaves. The results in this study indicate a correlation between the accessibility of glucan to water and to enzymes.

**Conclusions:**

Enzymatic degradability of wheat straw anatomical fractions can be indicated by the accessibility of the hydroxyl groups to water. This suggests that water may be used to assess glucan accessibility in biomass samples.

## Background

Diminishing supplies of fossil fuels and increasing greenhouse gas emissions call for sustainable energy resources. Wheat biomass residue is believed to be a potential feedstock for second-generation biofuel [1]. However, significant progress is needed to reduce processing costs and increase production efficiency [2]. One decisive factor is to understand and overcome the recalcitrance of the feedstock [3]. An important factor in recalcitrance for a given plant species is the type of tissues and cells present, and this differs substantially between different anatomical parts of the plant. The importance of the anatomical fractions in lignocellulosic biomass has been addressed by several research groups [4-6]. This study focuses on the two major fractions of wheat straw, leaf and stem, and introduces a novel way of probing the recalcitrance of these two types of wheat straw biomass fractions before and after hydrothermal pretreatment by the use of interaction with water in either liquid or vapor form.

Leaf and stem are the two major anatomical fractions of wheat straw. On weight basis, the leaf constitutes 25 to 50% and the stem 45 to 70% of the above-ground plant. Each wheat leaf is composed of blade, ligule, auricle and sheath [7], of which the blade and sheath are the major components by weight ratio. Mesophyll tissue, composed of homogeneous parenchyma cells, constitute the main part of the leaf interior. The leaf is coated by a dense epicuticular wax-rich epidermis [7]. Similarly, stem, composed of internodes and nodes also has waxy dermal tissues but lacks the large bulliform cells found at the upper epidermis of the leaf [7]. Sclerified parenchyma cells, embedded with vascular bundles, are the primary cells in stem internodes [6,7]. Between the epidermis and the parenchyma cells a sclerenchyma sheath is found, featuring several layers of cortical cells. The cuticle and epicuticular wax in the epidermis, lignified vascular bundles, and thick sclerenchyma fibers in stems contribute to the recalcitrant nature of wheat straw as summarized by Himmel *et al*. [3]. The differences in structural and chemical features of these two fractions lead to different performance during pretreatment and enzymatic hydrolysis [5,8].

Duguid *et al*. reported the distribution and composition, examined the effects of acid and alkaline pretreatment and compared the hydrolysis efficiency of four major anatomical fractions from wheat straw [4]. Chaff was most susceptible to pretreatment and hydrolysis, and nodes and internodes were the most recalcitrant fractions. The impact of leaf-to-stem (L/S) ratio on the enzymatic hydrolysis process was investigated by Zhang *et al*. for nine winter wheat cultivars by comprehensive microarray polymer profiling (CoMPP) for characterization and a high-throughput screening platform for enzymatic hydrolysis [5]. Increasing the L/S ratio resulted in the release of more sugar in moderate hydrolysis conditions and pure leaves yielded up to 30% more glucose than pure stems [5]. Another recent study compared the effects of laboratory- and industrial-scale hydrothermal (HT) pretreatment methods on winter wheat straw leaves and stems [6]. Rather than the degree of tissue disruption, the fraction of parenchyma cells turned out to be the most critical factor in wheat straw digestibility [6].

HT pretreatment is considered a promising method for industrial production. It applies only water and heat during processing, which avoids handling and recycling chemicals at either acidic or alkaline conditions, minimizing equipment corrosion. The HT process relocates lignin and cleaves O-acetyl and uronic acid substitutions on hemicelluloses, thus generating acetic acid, which partly hydrolyzes the hemicellulose during the pretreatment [9]. HT pretreatment has been reported to remove hemicelluloses and increase cellulose accessibility, but it has little or no effect on lignin removal and cellulose crystallinity [10,11]. Lignin relocalization occurs at temperatures above 150°C, which improves cellulose accessibility [12,13]. In this study, a pilot-scale HT pretreatment setup was used to treat wheat straw leaf and stem separately for further investigations of chemical composition, enzymatic digestibility, crystallinity and cellulose accessibility.

Recent studies indicate that the digestibility of lignocellulosic biomass is linked to water structuring [14,15]. These studies have employed low field nuclear magnetic resonance (LF-NMR) to map different states of water in lignocellulosic materials and to probe cellulose-water interactions. The spin-spin (T_2_) relaxation times measured using LF-NMR depends on the environment of the hydrogen nuclei in the sample. More tightly bound water gives shorter T_2_ values, whereas free water has longer spin-spin relaxation times. For plant tissues T_2_ values also reflect the lumen sizes of water-filled cells [16]. Felby *et al*. applied LF-NMR to describe different water locations during enzymatic hydrolysis of filter paper and how the water states and locations changed during enzymatic saccharification [17].

Another relevant technique may be Fourier transform infrared (FT-IR) spectroscopy to measure the level of water-accessible hydroxyl groups in the lignocellulosic matrix by hydrogen-deuterium exchange. This has previously been applied to monitor the hydrogen-deuterium exchange in accessible hydroxyl groups of celluloses [18-20]. However, to the best of the authors’ knowledge, this method has not been applied to probe the accessibility of biomass and how it may be connected to enzymatic hydrolysis. FT-IR absorbance in the 3,700 to 3,000 cm^−1^ range is due to hydroxyl stretching. The environment in which the hydroxyl bond is situated (that is, primarily the extent of hydrogen bonding) determines the exact position of the absorbance band [19-21]. It is known that the specific locations of hydroxyl stretching bands in the 3,500 to 3,300 cm^−1^ range are translated about 1,000 cm^−1^ towards lower wavenumbers or by a ratio of 1.34 upon deuteration [19,21]. This study used TD-NMR and FT-IR of deuterated samples to probe glucan accessibility and the impact of HT pretreatment on wheat straw leaves and stems with the aim of exploring to what extent the differences observed in degradability between wheat stems and leaves relate to hydration and the states of water present. More traditional measures of recalcitrance, that is, xylan removal and cellulose crystallinity were also examined within the frame of understanding differences in degradability.

## Results and discussion

### Wheat straw anatomical fractions

The distribution of major wheat straw anatomical fractions is shown in Table 1 for a set of twenty-one cultivars grown the same year at the same location. Leaf is mainly composed of leaf sheath because of loss of leaf blades while harvesting. The L/S ratio, which was previously found to be an important parameter during enzymatic conversion [5], varied from 0.42 to 1.00 within the sample set (Table 1). Stem nodes and internodes constituted between 48 and 68% of the wheat straw biomass (Table 1). Lindedam *et al*. reported that the overall sugar yield of wheat biomass could be affected by numerous factors including cultivar types, site-specific growth conditions, and chemical composition [8]. When studying the L/S ratio, Lindedam *et al*. were unable to find significant correlations with wheat biomass digestibility [8]. The authors suggested that efficient pretreatment may shield or weaken the actual differences between leaf and stem digestibility [8]. Additionally, the term leaf was confined to leaf blade by Lindedam *et al*., which contributed to no more than 11% (average) of the whole biomass by weight ratio [8]. Compared to our study where leaf sheath and blade contributed up to 48% of the whole wheat plant weight (Table 1), it becomes clear that the more inclusive definition of leaf will lead to a much larger fraction, which consequently may have a larger impact on wheat digestibility.

**Table 1:** Distribution of major anatomical fractions of twenty-one winter wheat straw cultivars

	**Leaf**^**a**^**%**	**Stem**^**b**^**%**	**Other%**	**Leaf-to-stem ratio**
*Robigus*	48	48	4	1.00
*SJ05-20*	43	50	7	0.86
*Legron*	36	62	3	0.58
*Proventus*	35	61	5	0.57
*Manager*	32	65	2	0.49
*PBI00373*	37	56	7	0.66
*Ellvis*	33	65	2	0.51
*MH0520*	30	68	1	0.44
*Portland*	36	62	2	0.59
*LP5184.4.0*	42	56	2	0.76
*LP227.1.03*	39	56	5	0.68
*Tritex*	35	60	5	0.59
*19429.28*	31	67	5	0.47
*NSL_01_5071*	34	61	4	0.56
*Cassiopeia*	42	54	4	0.77
*Paj702-406C*	41	54	5	0.77
*NIC00-3300A*	26	63	11	0.42
*Schamane*	36	62	2	0.58
*CE0412*	39	52	9	0.75
*Melkior*	38	59	3	0.64
*SW52747*	46	53	1	0.88
Average	37 (5)	59 (5)	4 (2)	0.65 (0.15)
Max	48	68	11	1.00
Min	26	48	1	0.42

Samples were harvested at Tystofte, Denmark in 2006. Numbers in parentheses are standard deviation (n = 21). ^a^Leaf = leaf blade and sheath; ^b^Stem = culm nodes and internodes.

The chemical compositions of untreated and HT-treated *Robigus* leaves and stems are shown in Table 2. The chemical composition results of liquid fractions of both HT-treated *Robigus* leaf and stem showed the presence of a trace amount of sugars. Untreated leaves and stems had very similar xylan contents, whereas the stems were richer in glucan and lignin, and the leaves contributed relatively more arabinan, galactan and ash (Table 2). In a recent study wheat leaf blade was demonstrated to contain less xylan and lignin, but similar glucan content compared to wheat stalk (leaf sheath and stem) [6]. After HT pretreatment the stems had higher glucan content (46%) compared to leaves (41%), similar lignin content, and much lower ash content (1%) than leaves (5%) (Table 2). The applied HT pretreatment method used in this study showed quite poor xylan removal efficiency (Table 2) compared to the industrial process [13]. However, the cell wall structure may still have been altered favorably for enzymatic hydrolysis as more xylan was released from the HT-treated samples than from the untreated samples during the subsequent enzymatic hydrolysis (Table 3). The composition of this type of HT pretreated wheat straw samples has been analyzed earlier [6,13]. It was reported that the xylan content of wheat straw dropped significantly from 24.5 to 5.2% after pretreatment [13]. In comparison, the glucan content of both HT treated leaves (41%) and stems (46%) were lower, in agreement with the poor xylan removal (Table 2).

**Table 2:** **Proportion of tested compounds in untreated and HT-treated *Robigus *** **leaves and stems**

	**Component%**						
	**Arabinan**	**Galactan**	**Glucan**	**Xylan**	**Lignin**	**Ash**	**Mass balance**
Untreated leaf	3 (0.2)	1 (0.0)	35 (1.0)	21 (0.9)	19 (1.2)	9	88
Untreated stem	2 (0.1)	1 (0.0)	41 (0.3)	21 (0.5)	22 (0.7)	5	92
Treated leaf	2 (0.1)	1 (0.0)	41 (1.1)	18 (1.0)	28 (1.2)	5 (0.6)	95
Treated stem	1 (0.0)	-	46 (0.6)	21 (0.5)	27 (0.5)	1 (0.1)	96

Data presented as mean value of triplicates. Standard deviations are given in parentheses (n = 3).

'–' indicates the compound is not detected.

**Table 3:** **Glucose and xylose yield of *Robigus *** **leaves and stems**

	**24-h Hydrolysis**		**72-h Hydrolysis**	
	**Glucose yield**^**a**^	**Xylose yield**^**a**^	**Glucose yield**	**Xylose yield**
Untreated leaf	0.33 (0.04)	0.09 (0.01)	0.32 (0.03)	0.11 (0.01)
Untreated leaf + xylanase	0.38 (0.01)	0.30 (0.01)	0.43 (0.03)	0.34 (0.02)
Untreated stem	0.19	0.12	0.25 (0.03)	0.10 (0.01)
Untreated stem + xylanase	0.24 (0.01)	0.20 (0.01)	0.26 (0.03)	0.22 (0.02)
HT leaf	0.36	0.53 (0.01)	0.53	0.66
HT leaf + xylanase	0.47 (0.02)	0.68 (0.06)	0.53 (0.06)	0.66 (0.08)
HT stem	0.33 (0.01)	0.53 (0.01)	0.33 (0.05)	0.44 (0.07)
HT stem + xylanase	0.36 (0.02)	0.60 (0.03)	0.46 (0.02)	0.61 (0.03)

Enzymatic hydrolysis condition: 5% dry matter, 5 filter paper units (FPU) g^−1^ dry matter, 800 rpm, 72 h. Reaction volume: 2 mL. Data presented as mean value of triplicates. Standard deviations are given in parentheses (n = 3). Standard deviations are not stated if the values are lower than 0.001. ^a^Glucose yield and xylose yield were calculated by the ratio of determined sugar concentration to the theoretical sugar content. HT, hydrothermally treated.

### Enzymatic digestibility

Untreated and HT-treated *Robigus* leaf and stem fractions were enzymatically hydrolyzed to compare the digestibility and the effect of HT pretreatment on these two fractions. Overall, wheat straw leaf was more digestible than the stem as shown in Table 3. Untreated leaves released up to 14% more glucose than untreated stems, and the HT-treated leaves yielded 20% more glucose in comparison with the stems after 72 h of hydrolysis (Table 3). Comparing untreated leaf with HT-treated leaf, the glucose yields were almost the same (<3% difference) after 24 h of hydrolysis, although much more xylose was produced from HT-treated leaf (53%) than from untreated leaf (9%). However, after 72 h of hydrolysis, 17% more glucose was released from HT-treated leaf, whereas no further glucose was produced from untreated leaf. This result confirms that HT pretreatment partially breaks down xylan, which gives the enzymes access to more cellulose [3,11], but it also shows that for the main part of the cellulose in leaves (*easy* glucan) this pretreatment is not needed and has no effect. This study shows that this is the case not only for the leaf blade but also for the leaf sheath. In stems slightly more glucose (6%) was produced from untreated material after 72 h compared to 24 h, but for HT-treated stem no further glucose was produced after 24 h.

**Figure 1:**
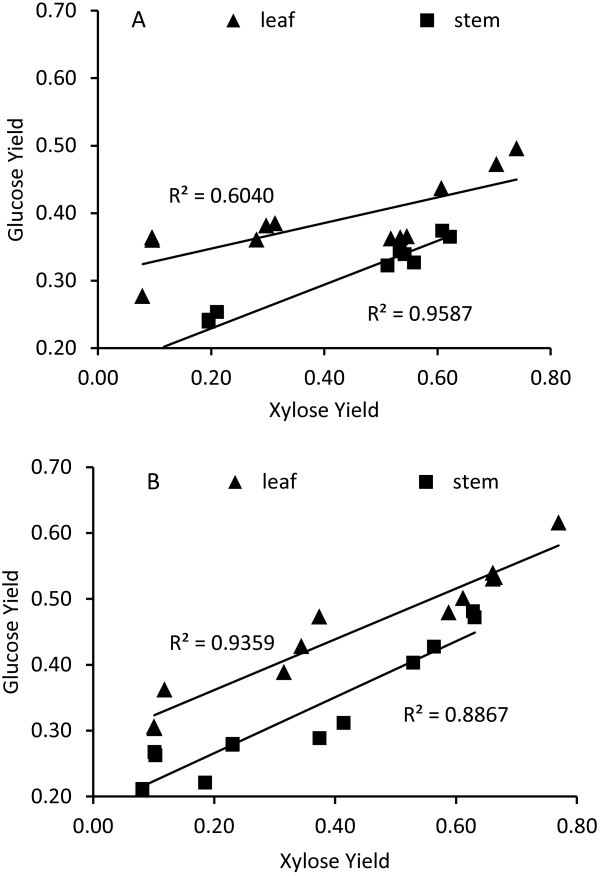
**Correlation between xylose and glucose yields of untreated and hydrothermally (HT)-treated *Robigus *leaves and stems. (A)** 24-h hydrolysis. **(B)** 72-h hydrolysis. Enzymatic hydrolysis conditions: 5% dry matter (DM), 5 filter paper units (FPU) g^−1^ DM, 800 rpm. Sugar yield was calculated as the ratio of released sugar to theoretical sugar (g/g).

Xylan removal or deacetylation of the xylan backbone has been demonstrated to substantially improve the biodegradability of many types of lignocellulosic biomass [22-26]. It is still under debate whether cross-linked cell wall matrix breakdown, xylan removal, or deacetylation of xylan backbones are the key factors in the biomass digestibility enhancement observed. Incomplete hydrolysis of xylan releases xylo-oligosaccharides and xylobiose or xylotriose during enzymatic hydrolysis, which inhibits cellubiohydrolase I (CBH I) competitively [27]. Also, soluble xylo-saccharides may change the distribution of water on the solids surface by lessening the water constraint at high solids saccharification [15]. Previously, Zhang *et al*. demonstrated that at very high enzyme loading, complete glucan hydrolysis was obtained on wheat straw leaves in 24 h, but complete xylan removal from the stems could only release 63% glucose [5]. The different performance under harsh enzymatic saccharification conditions may be due to the more recalcitrant nature of stem cellulose or the lower accessibility of cell wall glucans in stems compared to leaves. In this study additional xylanase and xylosidase enzymes increased the xylose yield from 9 to 30% for untreated leaves and from 12 to 20% for untreated stems, which resulted in higher glucan conversions, from 33 to 38% for the leaves and from 19 to 24% for the stems (Table 3). The correlation between glucose yield and xylose yield is summarized in Figure 1. More glucose was released from both untreated and HT-treated stems along with elevated xylose yield after both 24 h (*R*^2^ = 0.9587) and 72 h (*R*^2^ = 0.8867) of hydrolysis (Figure 1). The hydrolysis data for leaves showed a similar tendency as for stems, but the correlation was weaker after 24 h hydrolysis (*R*^2^ = 0.6040) (Figure 1A). These results indicate that both leaf and stem tissues are more easily degraded if extra xylanase activity is added, but that the effect is more pronounced for stems.

The different responses towards enzymatic hydrolysis from *Robigus* leaf and stem indicated: 1) HT pretreatment increased both xylose and glucose yields of leaf and stem fractions; 2) hemicellulose removal is more important for stem than leaf; and 3) by nature, leaf is less resistant to cellulosic enzymes than stem. Leaf sheath had a chemical composition similar to stem, but performed more like leaf blade during enzymatic hydrolysis, indicating that bulk chemical composition cannot stand alone as an indicator of recalcitrance. More basic investigations are needed to characterize the structural features of leaf sheath, which can contribute up to approximately 40% of wheat straw biomass by weight ratio.

### Recalcitrance

**Figure 2:**
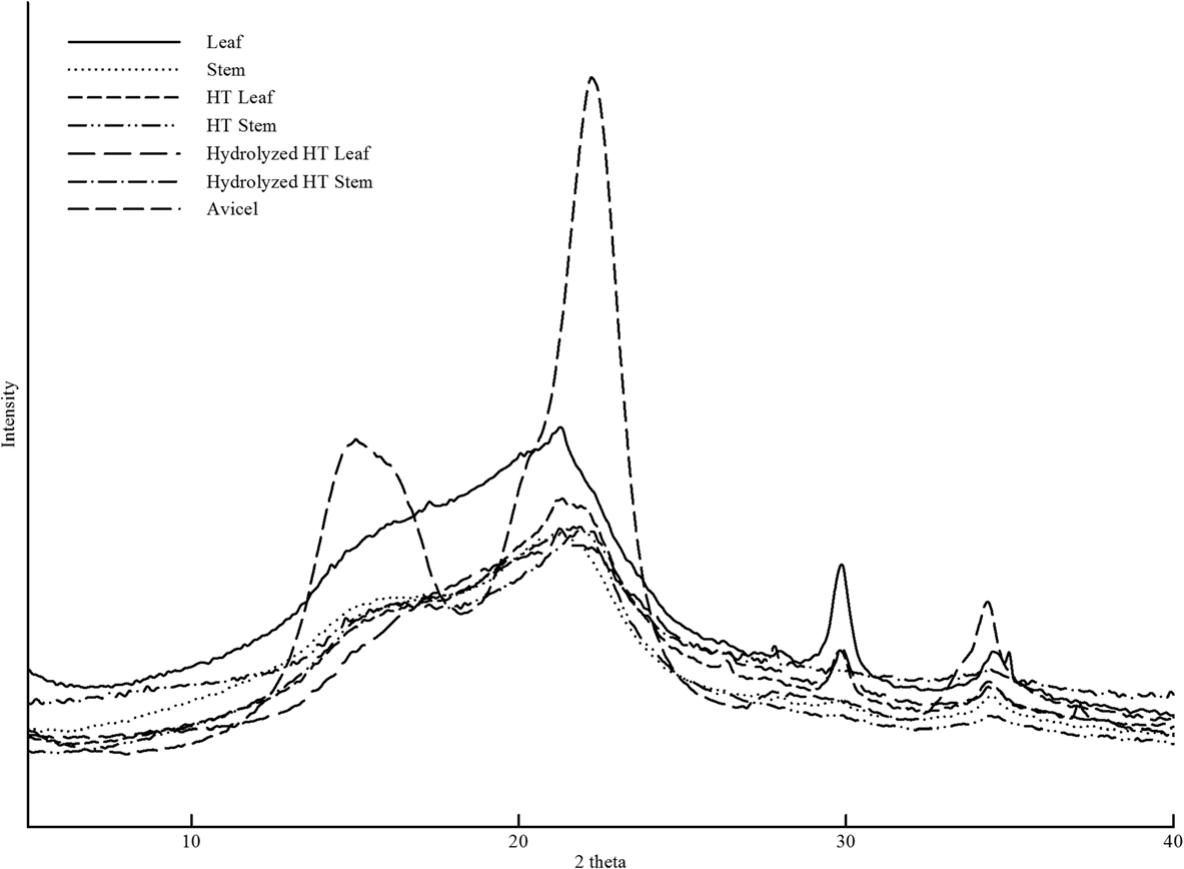
**WAXD curves of untreated, hydrothermally (HT) treated and enzymatically hydrolyzed *Robigus *leaves and stems.** 2 theta is the scattering angle.

For Avicel, cellulose crystallinity has been found to be linearly proportional to initial hydrolysis yield [28]. Cellulose crystallinity was also reported to affect the enzymatic hydrolysis of corn stover in the initial phase [29]. Crystallinity index (CI) values of cellulose extract from different wheat straw tissues were measured by Liu *et al*. using the crystalline area method [30]. The CI values of all wheat tissues (leaf sheath, epidermis, parenchyma and node) were similar and fairly low (approximately 40%) [30]. In this study, the relative CI values of untreated and HT-treated wheat straw (cultivar *Robigus*) leaves and stems and the change after hydrolysis were measured (Table 4). Wide angle x-ray diffraction (WAXD) curves of wheat straw leaves and stems are shown in Figure 2 and CI values are presented in Table 4. CI values of wheat straw leaves and stems samples were determined without extraction, in order to compare the relative cellulose crystallinity in the state subjected to enzymatic hydrolysis. As shown in Figure 2, at least four crystalline peaks (101, 10-1, 002 and 040) could be distinguished from the Avicel spectrum. The CI values of Avicel were 79.3 and 60.4, according to the peak height method and the peak deconvolution method, respectively (Table 4). However, the leaves and stems did not show clear peaks in the 2 *θ* range of 15 to 22°, but gave very broad peaks centered at 2 *θ* = 21.5^°^ (Figure 2). Technically, it is difficult to find the minimum position in between the 002 and 101 peaks from the leaves and stems. Therefore, the CI values according to the peak height method can only be used for comparison among the samples included in the present study. The CI values of untreated leaves (53.0) and stems (45.0) are not significantly different from each other when the standard deviations are taken into account (Table 4). Thus, the WAXD results corroborate earlier findings that wheat straw cellulose crystallinity does not seem to vary among different organ or tissue types. Neither wheat straw leaves, nor stems obtained different CI values after HT pretreatment (Table 4). The enzymatic hydrolysis results in this study showed that wheat straw leaves, both untreated and HT-treated have higher convertibility than stems. Consequently, our data do not support a correlation between the digestibility of wheat straw anatomical fractions and CI values, either before or after HT pretreatment.

**Table 4:** **Crystallinity index (CI) of untreated, HT treated and enzymatically hydrolyzed *Robigus *** **leaves and stems**

**Sample**	**Peak height**	**Peak deconvolution**
Untreated leaf	33.5 (5.2)	53.0 (6.9)
HT-treated leaf	37.9 (1.04)	43.5 (1.7)
Hydrolyzed HT leaf	33.5 (5.27)	45.0 (0.7)
Untreated stem	30.5 (0.97)	45.0 (1.2)
HT-treated stem	28.5 (0.53)	46.8 (1.3)
Hydrolyzed HT stem	52.6 (5.4)	39.9 (7.4)
Avicel	79.3 (0.08)	60.4 (1.2)

Numbers in parentheses are standard deviations (n = 3). HT, hydrothermally treated.

**Figure 3:**
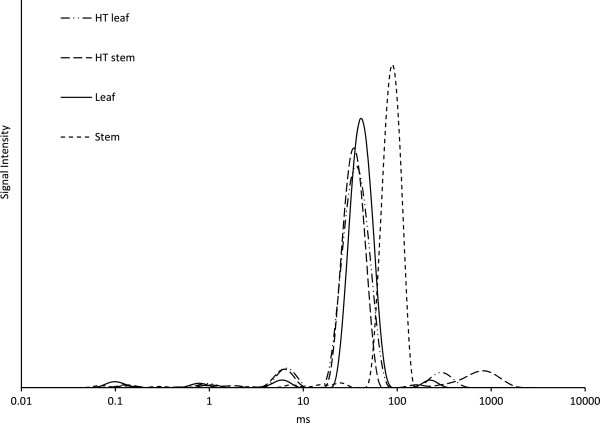
**Low field nuclear magnetic resonance (LF-NMR) T**_**2 **_**relaxation time distributions of untreated and hydrothermally (HT) treated *Robigus *** **leaves and stems.**

LF-NMR and FT-IR were employed to analyze and compare biomass-water interactions for stems and leaves. Figure 3 shows T_2_ relaxation time distributions of both untreated and HT-treated wheat straw leaves and stems. The major water peak in untreated stems is seen at 85 ms, while the corresponding peak for untreated leaves is located at 35 ms. The peaks in this range can be assigned to water in the lumens of differently sized cells and to interstitial water between particles [31], with longer relaxation time corresponding to larger cell lumens. The peaks at 0.1, 0.7 and 5 ms most likely refer to more tightly bound water situated in the cell wall [17]. Surprisingly, none of these peaks were found in untreated stems (Figure 3), which indicates easier wetting of leaf cell wall compared to stem cell wall, in accordance with the leaves being more easily degradable by the enzymes. The applied HT pretreatment influenced the state of water more significantly in stems than in leaves: Two peaks emerged at around 5 ms and 1,000 ms and the lumen water peak shifted to a shorter T_2_ for the stems, whereas the T_2_ distribution was almost unchanged for leaves (Figure 3). In other words, the spin-spin relaxation time distribution for stems was altered by the HT pretreatment so that it resembled that of leaves, which on the other hand was largely unchanged by the pretreatment. This observation is in line with the hydrolysis results, which showed that mainly stems benefitted from the pretreatment.

**Figure 4:**
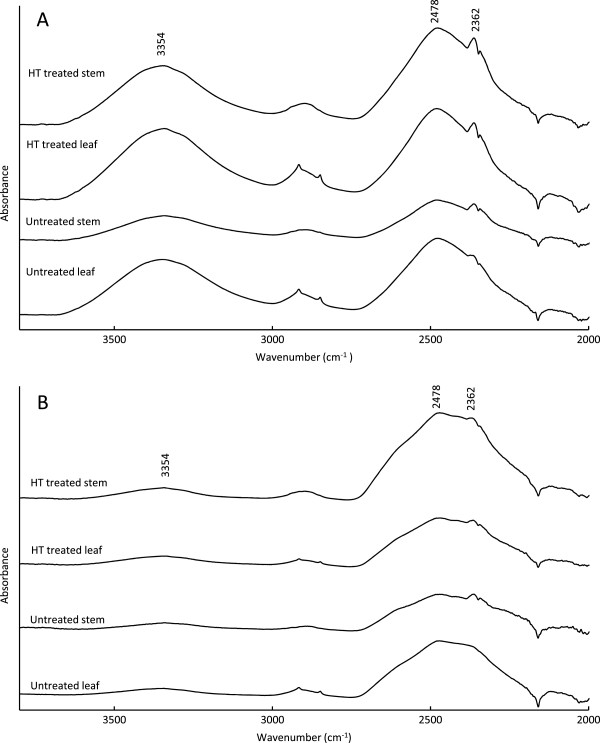
**Fourier transform infrared (FT-IR) spectra of hydrogen-deuterium exchange of untreated and hydrothermally (HT) treated *Robigus *leaves and stems. (A)** 20 h incubation time. **(B)** 240 h incubation time.

For deuteration all samples were incubated above liquid water at room temperature for 240 h, and then placed above deuterium water for either 20 or 240 h. FT-IR results indicated that water vapor exchanged more easily in wheat straw stems compared to leaves (Table 5), as more Oxygen-hydrogen (OH) stretching had been replaced by Oxygen-deuterium (OD) stretching vibrations in the stems after 20 h. Virtually no effects of HT treatment were observed (Table 5). After 240-h incubation, the peak area ratios (AR) for both wheat straw leaves and stems indicated nearly complete deuterium exchange (Table 5). Previously, the role of different hydrogen bonds within cellulose was demonstrated by several research groups [18,19,21]. According to these studies, the band at 2,478 cm^−1^ can be assigned to O (3) D…O (5) intramolecular bonds, whereas the 2,362 cm^−1^ relates to O (3) D…O (6) intermolecular bonds, that is, hydrogen bonds between cellulose chains [18]. Figure 4A and 4B show FT-IR spectra after 20 h and 240 h of deuteration, respectively. All samples showed a broad band at 2,478 cm^−1^. Some samples also absorbed at 2,362 cm^−1^. Although peak areas did not differ much between leaves and stems or before and after HT pretreatment (Table 5), small differences in the OD-stretching region were observed. These might show differences in the accessibility of glucan to water vapor. Intensity ratio (IR), the ratio of peak intensity at 2,478 cm^−1^ to 2,362 cm^−1^, was calculated and provided information on the distribution of deuterium exchange locations. Higher IR values were obtained from leaves than stems after 20 h deuterium exchange time (Table 5) suggesting that the least accessible class of hydrogen bonds in leaf were more accessible than the corresponding bonds in stem. The observed spectral differences are small, but that degradability and hydration may be linked is in line with the hydration experiments carried out by Selig *et al*. according to which the hydration of different cellulose crystal allomorphs (I, II and III) was related to their biodegradability [32].

**Table 5:** **Hydrogen-deuterium exchange of untreated and hydrothermally (HT) treated *Robigus *** **leaves and stems**

**Sample**	**Area ratio (AR)**		**Intensity ratio (IR)**	
	**20 h**	**240 h**	**20 h**	**240 h**
Untreated leaf	0.59	0.92	1.26	+∞
Untreated stem	0.65	0.90	1.13	0.98
HT-treated leaf	0.58	0.88	1.11	1.06
HT-treated stem	0.62	0.91	0.89	1.07

Data presented as the average of four replicates. AR is the ratio of peak area at OD-stretching (2,800 to 2,159 cm^−1^) to the total hydroxyl-stretching (2,800 to 2,159 cm^−1^ and 3,700 to 3,000 cm^−1^). IR is the ratio of peak intensity at 2,478 cm^−1^ to 2,362 cm^−1^.

**Figure 5:**
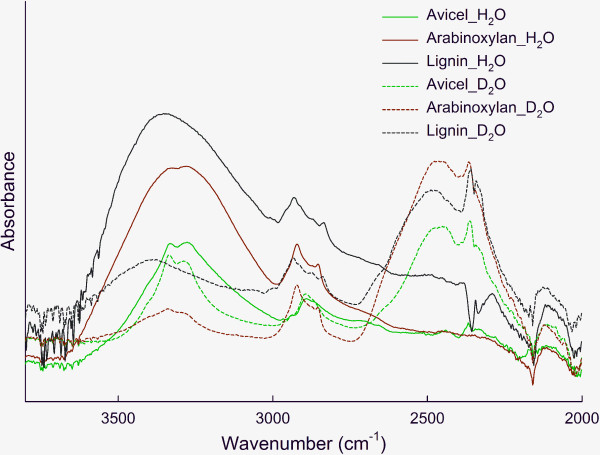
**Fourier transform infrared (FT-IR) spectra.** Avicel, wheat arabinoxylan and lignin were incubated above water or deuterium water for 20 h.

The samples in the present study are plant biomass samples and also contain hemicelluloses and lignin, both of which also have hydroxyl groups, and direct comparison to results for isolated cellulose is consequently not valid. However, we hypothesize that the 2,478 cm^−1^ band can be assigned to less accessible hydroxyl groups than the 2,362 cm^−1^ band also for biomass samples containing multiple types of hydroxyl groups. A supplementary experiment on Avicel, wheat arabinoxylan and alkali lignin demonstrated the water/heavy water vapor exchange of these model compounds when exposed to either water or heavy water for 20 h (Figure 5). After heavy water vapor exposure, Avicel and wheat arabinoxylan showed identical patterns in the OD region, that is, two distinguishable peaks at 2,476 cm^−1^ and 2,362 cm^−1^, in line with observations on cellulose [18,19,21] and our observations on wheat straw biomass (Figure 4). Lignin showed absorbance bands at 2,490, 2,359 and 2,343 cm^−1^, that is, partially overlapping with the glucans. A weak band at 2,343 cm^−1^ was also observed in the wheat straw samples (Figure 4), indicating that H/D exchange to some extent also took place in lignin. In comparison with Avicel samples, wheat arabinoxylan and lignin absorbed and exchanged water more rapidly, suggested by the stronger absorbances for both HO and DO; in other words, hydroxyl groups in arabinoxylan and lignin were more accessible than in Avicel (cellulose). However, it is risky to conclude from these observations anything about the ease or difficulty of hydration of the different polymers when built into the biomass, as the cell wall structure may give rise to swelling resistance and consequently reduced accessibility to binding sites, which is not the case for the isolated components. We hypothesize that the observation of more water-accessible hydroxyl groups in leaves compared to the corresponding hydroxyl groups in the stem translates into higher accessibility for cellulases. However, further insight into the interactions between water-enzyme-lignocellulose is needed.

## Conclusions

This study confirmed earlier findings that wheat straw leaves give higher glucose and xylose yields compared to stems during enzymatic hydrolysis. A large variation in L/S ratio among wheat straw cultivars was found and might be utilized to improve wheat biomass digestibility by choosing cultivars with high L/S ratios. It was also confirmed that hemicellulose removal can significantly improve the digestibility of wheat straw feedstocks, and that this relationship is stronger for stems than for leaves. No difference in crystallinity between leaf and stem was found, but LF-NMR and FT-IR indicated that glucans are more accessible to water in leaves than in stems, both in liquid and in vapor form. Together with enzymatic hydrolysis yields this result suggests that water accessibility is linked to biodegradability and can be used as an indicator for recalcitrance.

## Methods

### Wheat straw sample preparation

A set of 21 winter wheat cultivars grown under the same conditions and site in Denmark were fractionated to investigate the naturally occurring variation in the L/S ratio (Table 1). Wheat samples were harvested from the statutory field trails in Tystofte, Denmark in 2006 and kept in sealed plastic bags at ambient temperature until analysis. Each cultivar was fractionated by hand into two fractions: leaf (leaf blade and leaf sheath) and stem (internodes and nodes) and weighed. The *Robigus* cultivar was chosen for study in further experiments due to the fact that this cultivar has the highest L/S ratio among the tested 21 cultivars (Table 1). This ensured that there was enough leaf biomass for separate HT pretreatment using the Integrated Biomass Utilization System (Mini IBUS) equipment at the Technical University of Denmark (Risø Campus). Wheat straw leaves and stems were processed separately at an initial dry matter (DM) content of 40% at 190°C for 10 minutes by steam followed by a pressing step. After pretreatment both liquid and solid fractions were kept at −20°C until use.

Untreated wheat leaves and stems were air-dried; HT-treated samples were washed in distilled water and then oven dried at 40°C for approximately two days. All samples were ball-milled using a Planetary Ball Mill PM 400, Retsch with four grinding stations.

### Compositional analysis

The DM content of air dried, ball-milled wheat samples, both untreated and treated was determined using a Sartorius MA 30 moisture analyzer at 105°C. The composition of carbohydrates and lignin was determined using a modified Klason lignin method derived from the TAPPI standard method T222 om-98 (TAPPI 5960). Briefly, 0.3 g of dried sample was incubated with 3 mL of 72% H_2_SO_4_ for 1 h at 30°C with mixing. The sample was then diluted with deionized water to a final acid concentration of 4%. The solution was autoclaved for 1 h at 121°C and filtered through a medium-coarseness sintered-glass filter for gravimetric determination of acid insoluble lignin. Each sample was analyzed in triplicate. The concentration of sugars in the filtrate was determined by HPLC with quantification referenced to standards, which were also autoclaved in 4% H_2_SO_4_ to compensate for degradation.

Monosaccharides from compositional analysis (arabinose, galactose, glucose, mannose and xylose) were determined using an ICS 5000 system from Dionex (Sunnyvale, California, USA). The separation was performed in a Dionex CarboPac PA1 column at 30°C with a flow rate of 1 mL min^−1^ of MilliQ (MQ)-water and using fucose as the internal standard. Detector sensitivity was optimized by post-column addition of 0.2 M NaOH at a flow rate of 0.5 mL min^−1^. The column was cleaned after each sample with 0.25 M NaOH for 5 minutes and then reconditioned by MQ water for 5 minutes. The samples were diluted in MQ-water, mixed with the internal standard and filtered through a 0.45-μm nylon filter before injection.

### Enzymatic hydrolysis

Enzymatic hydrolysis were performed on both untreated and HT-treated samples at a solids loading of 5% DM and an enzyme loading of 5 FPU (filter paper units) g^−1^ DM. This was achieved by dispensing 0.1000 g DM of *Robigus* leaves and stems into 2-mL capped tubes and adding 1.8 mL 50-mM sodium citrate buffer (pH 5). Two enzymatic treatments were used, one with a cellulase mixture of Celluclast 1.5 L and Novozyme 188 in a weight ratio of 5:1 (Novozymes A/S, Bagsværd, Denmark), and one where the mixture was supplemented with extra xylanases. *Endo*-1, 4-β-Xylanase M1 (*Trichoderma viride*) and *exo*-1, 4-β-D-xylosidase (*Selenomonad ruminantium*) were used at 170 U g^−1^ DM and 30 U g^−1^ DM, respectively (Megazyme, Bray, Ireland). The enzymatic treatment with extra xylanases was only used in glucose and xylose yield comparison of *Robigus* leaves and stems (Table 3). Samples were incubated at 50°C, 800 rpm for 24 h or 72 h. Samples were filtrated through 0.45 μm plate filter and loaded to HPLC for sugar determination. Calculation of glucose and xylose yield was based on the theoretical carbohydrate content of untreated or HT-treated wheat straw leaves and stems.

Monosaccharides (D-glucose, D-xylose, and L-arabinose) released from enzymatic hydrolysis were measured on a Dionex Ultimate HPLC system (Dionex, Germering, Germany). The separation was done in a column at 80°C with 5 mM H_2_SO_4_ as eluent at a flow rate of 0.6 mL min^−1^ and quantified by an RI-detector. The samples were diluted in eluent and filtered through a 0.45-μm nylon filter before injection.

### Wide angle x-ray diffraction (WAXD)

The x-ray diffraction was performed using a Ganesha Instrument from SAXSLab/JJ-Xray, Denmark. The x-ray beam was monochromatic CuK_α_-radiation (x-ray wavelength *λ* = 0.154 nm) obtained from a Rigaku MicroMax-002 source equipped with an optical module composed of two perpendicular super mirrors and using electrostatic focusing. The source was operated with 42 kV and 0.95 mA. The collimation was obtained using a three pin-hole configuration, and the scattered x-rays were measured by a two-dimensional 300 K Pilatus detector from Dectris. Both the collimation parameters and the sample-to-detector distance can be changed within the Ganesha Instrument, for wide-angle or small-angle scattering regimes within the same instrument, thereby covering structural features from less than 0.2 nm to more than 200 nm. The x-ray data reported were obtained with entrance collimation pinhole equal to 0.7 mm, defining pinhole near the sample equal to 0.4 mm, and sample-to-detector distance equal 180 mm. Other configurations showed no structural features beyond surface scattering from the powder samples. The samples were mounted in sealed holders with two 5 to 7 μm thick mica windows and measured in a vacuum. The data analysis, including azimuthal averaging, was done using SAXSGui from SAXSLab, and the spectra are presented versus the scattering angle 2 *θ*. Many different methods exist for the calculation of CI, such as the peak height method [33], the deconvolution method [34], the amorphous subtraction method [35] and the NMR C4 peak separation method [36]. CI values vary with analytical method and calculation principles [34], but CI values are still applicable to compare the relative crystallinity level of different samples, provided that the same procedure is used throughout. In this study two calculation methods, peak height and peak deconvolution were used to determine the relative CI values of untreated, HT-treated and enzymatically hydrolysed wheat leaves and stems. OriginPro 9 software was applied to determine the peak height ratio and peak area ratio. It has been pointed out that the peak height method overestimates the CI and excludes all the other crystalline peaks besides the 002 peak in calculation, whereas the peak deconvolution method takes all observed crystalline peaks into consideration and calculates the CI by the ratio of the sum of the areas of the crystalline peaks to the total area below the curve [34].

### Low field nuclear magnetic resonance (LF-NMR)

NMR analyses were done using a Bruker mq 20-Minispec, with a 0.47 Tesla permanent magnet (20 MHz proton resonance frequency), operating at 40°C. The transverse (T_2_) relaxation times were determined using the Carr-Purcell-Meiboom-Gill (CPMG) sequence. About 3,000 echoes were collected with a pulse separation of 0.04 ms, the acquisition of 32 scans and a 5-s recycle delay. The CPMG relaxation curves were then analyzed using the inverse Laplace transformation method CONTIN [37] to obtain the T_2_ relaxation time distributions. Both milled untreated and treated wheat leaves and stems were dispensed in MQ-water to obtain 23% DM, and incubated at 40°C for an hour prior to the measurement.

### Fourier transform infrared spectroscopy (FT-IR)

FT-IR measurements were performed on ball-milled samples using a Nicolet 6700 spectrometer from Thermo Fisher equipped with a PIKE Diamond ATR unit. Spectra were obtained in the spectral range of 4,000 to 600 cm^−1^ using 64 scans, Happ-Genzel apodization, and a resolution of 4 cm^−1^.

Oven dried wheat straw samples (50 mg) were weighed into NMR tubes and incubated at room temperature in a desiccator above liquid water for 240 h for equilibration prior to hydrogen-deuterium exchange. For vapor exchange samples were then placed over deuterated water (99.9 atom% D) in another desiccator and measured by FT-IR after 20 h and 240 h.

Chemicals used in vapor exchange experiments on cell wall model compounds were commercial products. Avicel® PH-101 (11365 FLUKA) was purchased from Sigma-Aldrich, Denmark ApS. Insoluble wheat arabinoxylan was purchased from Megazyme, Bray, Ireland. Chemical composition includes arabinose, 36%; xylose, 51%; glucose, 6.5%; mannose, 4.4% and galactose, 1.6%. Alkali lignin (370959) was purchased from Sigma-Aldrich, Denmark ApS. OriginPro 9 software was applied to calculate the peak IR and peak AR.

## Abbreviations

AR: area ratio; CBH I: cellubiohydrolase I; CI: crystallinity index; CoMPP: comprehensive microarray polymer profiling; DM: dry matter; FPU: filter paper units; FT-IR: fourier transform infrared; HPLC: high performance liquid chromatography; HT: hydrothermal; HTP: high throughput; IR: iIntensity ratio; LF-NMR: low field nuclear magnetic resonance; L/S: leaf to stem; WAXD: wide angle x-ray diffraction.

## Competing interests

The authors declare that they have no competing interests.

## Authors’ contributions

HZ: study coordination, wheat sample dissection, compositional analysis, enzymatic hydrolysis, sugar measurements, LF-NMR and IR experiments, data collection and analysis, manuscript draft and revision. LGT: suggestion on the use of LF-NMR and FT-IR, support on LF-NMR and FT-IR experiments, critical manuscript revision and discussion. KM: WAXD experiments, technical support on data analysis, manuscript revision. ZK: support on pilot scale HT pretreatment, manuscript revision and discussion. JL: wheat straw samples supply, outlining the study, manuscript preparation. HJ: manuscript discussion and revision. CF: outlining the study, supervision, manuscript revision and final approval. All authors have read and approved the final manuscript.
